# Evaluation of the clinical outcome of hypercalcemia of malignancy and concurrent azotemia in dogs with lymphoma

**DOI:** 10.1111/jvim.16974

**Published:** 2023-12-22

**Authors:** Alyssa A. Strumpf, Laura Selmic, Brian Husbands

**Affiliations:** ^1^ The Ohio State University, College of Veterinary Medicine Columbus Ohio USA

**Keywords:** acute kidney injury, chemotherapy, dog, T‐cell lymphoma

## Abstract

**Background:**

Hypercalcemia of malignancy (HM) secondary to lymphoma in dogs has the potential to cause renal injury.

**Hypothesis/Objectives:**

Characterize outcomes related to acute kidney injury (AKI) secondary to HM. We hypothesized that dogs do suffer AKI regardless of HM severity at the time of lymphoma diagnosis or relapse.

**Animals:**

Retrospective study. Twenty‐nine dogs with lymphoma, HM, and azotemia (International Renal Interest Society [IRIS] grade II or higher AKI) that underwent chemotherapy were identified at 2 veterinary institutions.

**Methods:**

Logistic regression and descriptive statistical analysis were performed to evaluate data for potential prognostic factors.

**Results:**

After initiating treatment, resolution of hypercalcemia and azotemia occurred in 100% (29/29) and 79.3% (23/29) of dogs, respectively. Resolution of azotemia was influenced by serum creatinine concentration (odds ratio [OR], 0.148; Confidence interval [CI], 0.03‐0.734; *P* = .02) and total hypercalcemia (OR, 0.36; CI, 0.14‐0.93; *P* = .04) at diagnosis, whereas blood urea nitrogen concentration, IRIS grade, sex, and whether or not dogs were hospitalized were not significant factors. At data analysis, 13.8% (4/29) of dogs were alive or lost to follow‐up. Of those dead, 4 dogs (15%) had renal disease at the time of death, 2/4 having concurrent lymphoma progression.

**Conclusions and Clinical Importance:**

Although AKI may be of clinical concern in dogs with HM secondary to lymphoma at diagnosis, death secondary to renal impairment appears to be infrequent.

**Abbreviations**
ADH: anti‐diuretic hormone
AKI: acute kidney Injury
BUN: blood urea nitrogen
CKD: chronic kidney disease
GFR: glomerular filtration rate
HM: hypercalcemia of malignancy
IHC: immunohistochemistry
IQR: interquartile range
OST: overall survival time
PARR: PCR for antigen receptor rearrangement
PCR: polymerase chain reaction
PTHrP: parathyroid hormone related peptide
ROC: receiver‐operating characteristic
SD: standard deviation

## 1 INTRODUCTION

Paraneoplastic syndromes comprise a series of alterations associated with endogenous release of various hormones or an immune response by the tumor.^1^ Hypercalcemia of malignancy (HM), a paraneoplastic syndrome, predominantly occurs because of ectopic production of parathyroid hormone‐related peptide (PTHrP).^2^, ^3^, ^4^, ^5^ Severe hypercalcemia secondarily causes renal impairment by 4 mechanisms: decreased blood flow and glomerular filtration rate (GFR) secondary to vasoconstriction, impaired anti‐diuretic hormone (ADH) response, renal tubular damage caused by impaired calcium clearance, and volume depletion leading to tubular injury.^6^ Although unknown in the canine population, the incidence of HM is reported to be 10% to 30% in humans,^1^, ^3^ increasing dramatically with cancer progression.^1^ Various cancers are associated with HM in humans,^1^, ^7^ whereas in dogs, the principal cancers involved include lymphoma^8^ and anal sac apocrine gland adenocarcinoma, although others also have been reported.^2^, ^9^

In humans and dogs, HM reportedly carries a poor prognosis, resulting in substantial morbidity at the onset of disease secondary to its clinical complications, but, a recent study evaluating dogs with high‐grade primary mediastinal lymphoma did not find HM to be a prognostic indicator for outcome.^1^, ^8^ Previous reports documented acute renal failure associated with HM in humans diagnosed with cancer, and improvement in azotemia has been described with treatment.^10^, ^11^, ^12^ Unfortunately, these studies had small patient populations and lacked reported long‐term outcomes.^11^, ^12^ In dogs, literature describing whether mortality occurs as a result of acute kidney injury (AKI) or if treatment leads to resolution of this secondary sequela is limited.^13^ One publication reported short‐term assessments in azotemic dogs with HM secondary to lymphoma.^14^ Two out of 12 dogs had improvement in their azotemia 1 week after starting treatment, but long‐term outcomes were not reported. Another study described outcomes in dogs with mediastinal lymphoma and HM, of which 10 dogs were found to be azotemic at diagnosis.^8^ This study did not find significant differences in outcome between azotemic and non‐azotemic dogs, but information describing resolution of azotemia was not reported. There remains a paucity of information in dogs regarding long‐term outcome of HM secondary to lymphoma with concurrent azotemia.

Our primary aim was to characterize outcomes in dogs diagnosed with HM secondary to lymphoma and concurrent azotemia undergoing treatment. We aimed to characterize several outcomes, including resolution of HM, azotemia, lymphoma remission, and survival related to AKI secondary to HM. We hypothesized that dogs that develop AKI secondary to HM do not die from renal injury regardless of the severity of hypercalcemia or azotemia at the time of diagnosis or relapse. A secondary aim was to report renal changes detected with imaging at the time of diagnosis of lymphoma and the impact of these changes on outcome.

## 2 MATERIALS AND METHODS

### 2.1 Retrospective study

A bi‐institutional, retrospective study evaluating dogs diagnosed with intermediate‐ to high‐grade lymphoma, concurrent hypercalcemia and azotemia was performed. Cases were identified from the Veterinary Medical Centers at the University of Minnesota (UMN) and The Ohio State University (OSU). Medical records were searched from UMN between July 2004 and July 2019 and OSU between February 2007 and August 2021. Dogs were included if they had a cytologic or histologic diagnosis of intermediate‐ to high‐grade lymphoma, confirmed serum total or ionized hypercalcemia, International Renal Interest Society (IRIS) grade of AKI II or higher azotemia with inappropriate urine concentration at diagnosis (urine specific gravity [USG] < 1.030)^15^ and underwent chemotherapy. With a serum calcium concentration cut‐off of 12.0 mg/dL, suggested previous study found a 93% positive predictive value in accurately predicting ionized hypercalcemia in the presence of normophosphatemia in affected dogs.^16^ To further decrease the likelihood, we chose a minimum cut‐off of 12.5 mg/dL. Hypercalcemia was confirmed when either a second serum total calcium concentration was >12.5 mg/dL, or an ionized calcium concentration was above the normal reference range for the institution (5.4 mg/dL at OSU; 6.0 mg/dL at UMN). Azotemic dogs were included if they were found to have a serum creatinine concentration of at least 1.7 mg/dL, consistent with an IRIS grade II or higher AKI, with concurrent USG < 1.030.^15^, ^17^ Dogs with serum creatinine concentration < 1.7 mg/dL were excluded because IRIS grade I includes non‐azotemic dogs. Dogs were included if they underwent lomustine‐based chemotherapy, chemotherapy with cyclophosphamide, vincristine, prednisone (COP), or chemotherapy with cyclophosphamide, doxorubicin, vincristine, prednisone (CHOP) with an intended minimum follow‐up period of at least 3 months to allow time for resolution of hypercalcemia and azotemia or death secondary to AKI to occur unless they died or were euthanized because of lymphoma.

### 2.2 Review of medical records

Breed, sex, age, minimum lymphoma stage and substage, method of lymphoma diagnosis, immunophenotyping when performed, hospitalization at diagnosis, chemotherapy protocol, resolution or improvement and time to resolution of hypercalcemia and azotemia, recurrence of hypercalcemia at lymphoma progression, cause of death when known, necropsy findings when performed, and survival time were abstracted from medical records. Intermediate‐ to high‐grade lymphoma was definitively diagnosed by a board‐certified veterinary pathologist. Staging diagnostic tests performed at diagnosis were based on clinician preference and owner consent, and minimum stage of lymphoma was recorded using the World Health Organization staging scheme for multicentric lymphoma in dogs.^18^ Staging diagnostic tests included CBCs, serum biochemistry profile, serum ionized calcium concentration, urinalysis, cytology or histopathology of a lymph node or both, chest radiographs, and abdominal imaging. Dogs were not required to have complete staging for inclusion and were designated as minimum stage and substage based on history, physical examination, and initial diagnostic testing. Immunophenotype was determined by immunohistochemistry (IHC), flow cytometry, polymerase chain reaction for antigen receptor rearrangement (PARR), or some combination of these, when performed. Supportive care was at the discretion of the attending clinician and included a minimum of fluid therapy for dogs that were hospitalized. Glucocorticoids, other chemotherapy, and additional supportive care medications also were administered during hospitalization. For dogs with imaging performed at diagnosis, chest and abdominal radiographs and abdominal ultrasound imaging reports were reviewed by a board‐certified radiologist. Resolution of hypercalcemia and azotemia were characterized by results within the reference range for the contributing institution with reassessment of either total serum or ionized calcium and serum creatinine concentration, respectively. Repeat total calcium, ionized calcium, and creatinine concentration, or some combination of these was performed at the discretion of the attending clinician and timing was not standardized. Time to resolution of hypercalcemia and azotemia was defined as the number of days between lymphoma diagnosis and the date resolution was documented. Data regarding hypercalcemia and azotemia resolution were recorded after starting treatment at week 1 (3‐10 days after starting treatment), week 2 (11‐17 days after starting treatment), week 3 (18‐24 days after starting treatment), and week 4 (25‐31 days after starting treatment). Recurrence of hypercalcemia at relapse of lymphoma was recorded when available. Remission status, relapse of lymphoma, survival, and cause of death were determined from medical record review and, when lost to follow‐up, primary veterinarians were contacted for additional information. Survival time was calculated from the date of starting treatment until death. Dogs were censored if they were lost to follow‐up, death was unrelated to a non‐lymphoma related cause, or if they were alive at time of data analysis.

### 2.3 Statistical analysis

Descriptive statistics were calculated for continuous variables. The continuous variables were assessed for normality based on skewness, kurtosis, and Shapiro Wilk tests. Normally distributed variables were reported as mean and SD, whereas non‐normally distributed variables were reported as median with interquartile range (IQR). Univariable logistic regression was used to assess variables associated with resolution of azotemia. Variables assessed included sex, serum creatinine and blood urea nitrogen (BUN) concentration, IRIS AKI grade, total calcium concentration, and hospitalization at diagnosis. Likewise, logistic regression was used to assess renal ultrasound changes associated with disease‐related death. Cox proportional hazards analysis was used to assess the severity of hypercalcemia, serum creatinine concentration, and resolution of azotemia associated with survival time. Receiver operating characteristic (ROC) curve analysis was used to assess the range of serum total calcium and creatinine concentration, and their association with resolution of azotemia. Kaplan‐Meier analysis was used to calculate overall survival time (OST). Significance of data was measured at a *P* < .05. Statistical software (SAS 9.4; SAS Institute, Cary, North Carolina) was used in all analyses.

## 3 RESULTS

### 3.1 Patient population

Twenty‐nine dogs met the inclusion criteria. Presenting clinical signs varied and included polyuria, polydipsia, lethargy, vomiting, weight loss, hyporexia, or some combination of these signs.

Golden retrievers comprised 12/29 of the cases. The remaining breeds included mixed breed (4), Boxer (2), Shetland Sheepdog (2), and 1 each of the following: Dogue de Bordeaux, German Shepherd Dog, Bernese Mountain Dog, English Setter, Old English Sheepdog, Labrador Retriever, Collie, and Coonhound. Median age at diagnosis was 5.4 years (range, 2‐11 years). Neutered males comprised (18/29), intact males (2/29), and spayed females (9/29). Dogs were diagnosed with intermediate‐ to high‐grade lymphoma based on cytology (26/29) or a combination of cytology and histology (3/29). Based on the World Health Organization Staging Scheme,^18^ dogs were classified as having generalized intermediate‐ to high‐grade lymphoma in 16/29 cases and high‐grade thymic lymphoma in 13/29 cases. Of the thymic lymphoma cases, only 1/13 was thymic in origin without peripheral nodal involvement. There were no stage I dogs in this cohort; stage II represented 2/29 dogs, stage III 17/29, stage IV 8/29, and stage V 2/29. All dogs were characterized as substage b. Immunophenotyping based on IHC, flow cytometry, PARR, or a combination of these was performed in 20/29 dogs: IHC in 3/20, flow cytometry in 7/20, and PARR in 10/20. Of the combination immunophenotyping, 1/20 dogs had PARR and IHC performed and 1/20 had flow cytometry that was nondiagnostic followed by IHC. Of the 20 dogs with available phenotype, there were 19/29 T‐cell and 1/29 B‐cell (based on PARR and IHC). At the time of diagnosis, 66% (19/29) of dogs were hospitalized.

### 3.2 Hypercalcemia and IRIS grade AKI for dogs

Dogs (29/29) had confirmed increased total serum calcium or ionized calcium concentration. An increased serum total calcium concentration was documented in 28/29 dogs, with a mean of 15.1 ± 1.5 mg/dL (range, 12.5‐18.9). One dog had serum total hypercalcemia described in the medical record, but the numerical value was unavailable at data collection. This dog was found to have serum ionized hypercalcemia. Of the dogs with serum total calcium concentration available, 11/28 had another serum total calcium concentration repeated to confirm hypercalcemia. The remaining 18 dogs were found to have serum ionized hypercalcemia with a mean of 7.4 ± 0.8 mg/dL (range, 5.8‐8.8). International Renal Interest Society (IRIS) AKI grade II comprised 14/29, III 14/29, and IV 1/29 of the cases. The median serum creatinine concentration at diagnosis was 2.8 mg/dL (range, 1.7‐5.9; IQR, 2.0‐3.9). The median USG at baseline was 1.010 (range, 1.005‐1.022; IQR, 1.006‐1.014). There were 9/29 dogs with hyposthenuria (USG < 1.008), 12/29 with isosthenuria (USG 1.008‐1.012), and 8/29 dogs with minimally concentrated urine (USG 1.013‐1.029). Two isosthenuric dogs had been given fluids before collecting urine: 1 dog had a USG of 1.009 and had received 4 hours of fluid therapy before urine collection and the other had a USG of 1.012 and received fluids for approximately 12 hours at the referring veterinary practice the day before arrival at the referral hospital.

### 3.3 Baseline abdominal imaging

At diagnosis, 18/29 dogs had baseline abdominal imaging: ultrasound examination in 17/18 and radiography in 1/18. The abdominal radiographs were reported to be normal in this case. Because only 1 dog had abdominal radiographs performed, this dog was excluded from statistical analysis evaluating influence of renal imaging changes on outcome. Of the dogs having had abdominal ultrasonography performed, 7/17 had renal changes, with 5/7 showing a medullary rim sign, 1/7 having bilateral renal cortical mineralization, and 1/7 having mild bilateral pyelectasia.

### 3.4 Chemotherapy

All 29 dogs underwent chemotherapy after a diagnosis of lymphoma. Fourteen of 29 dogs were treated using a lomustine‐based protocol, 11/14 of which underwent a multi‐agent protocol (cyclophosphamide, lomustine, doxorubicin, and prednisone), and 1 each that underwent vincristine and lomustine, single‐agent lomustine, and a single dose of vincristine before transitioning to a lomustine‐based multi‐agent protocol. Two dogs underwent a COP protocol, 12 dogs underwent a CHOP protocol (25‐week or 19‐week), and 1 dog received cyclophosphamide, vincristine, cytosine arabinoside, and prednisone (COAP). All dogs received glucocorticoids. Fifteen of 29 dogs (52%) received L‐asparaginaseas first‐line therapy treatment.

### 3.5 Clinical outcomes—Hypercalcemia, azotemia, and lymphoma progression

Reassessment of hypercalcemia and azotemia was performed at the discretion of the attending clinician and the timing of reassessment was not standardized. Table 1 includes time to resolution of hypercalcemia and azotemia each week after starting treatment. All dogs had resolution of hypercalcemia, with times ranging from 1 to 68 days. Of these, 14/29 (48%) dogs had resolution of hypercalcemia within 7 days. Twenty‐three of 29 (79%) dogs had resolution of azotemia (mean ± SD, ±5.6), with times ranging from 6 to 334 days. Of these, 11/29 (38%) dogs had resolution of azotemia at >24 days. The remaining 6 dogs had improvement in their AKI grade but did not have resolution of their azotemia (Table 2). Of the 25 dogs documented to have lymphoma relapse, 13/25 (52%) did not have recurrence of hypercalcemia at the time of relapse, whereas 9/25 (36%) had recurrent hypercalcemia and 3/25 (12%) did not have serum calcium concentration reassessed. Recurrence or worsening of azotemia did not occur in 18/25 (72%) dogs at the time of lymphoma progression. Four of these dogs did not have resolution of their azotemia at the time of diagnosis. At the time of lymphoma progression, 2 of these dogs had stable azotemia from the time of initial diagnosis, whereas 2/25 (8%) dogs had worsening azotemia. At lymphoma progression, 5/25 (20%) dogs did not have repeat biochemistry profiles to screen for recurrence of azotemia. Two dogs with worsening azotemia at the time of lymphoma progression, both AKI grade III, were found to have recurrent hypercalcemia at lymphoma relapse.

**TABLE 1:** Time of resolution of hypercalcemia or azotemia in 29 dogs.

Clinical outcome data	Week one (3‐10 days)		Week two (11‐17 days)		Week three (18‐24 days)	
	Number of dogs	Percentage (%) resolved	Number of dogs	Percentage (%) resolved	Number of dogs	Percentage (%) resolved
Hypercalcemia	19	66	25	86	29	100
Azotemia	2	7	9	31	12^a^	41

^a^ The remaining 11 dogs had resolution of azotemia after 24 days (range, 25‐334 days).

**TABLE 2:** Initial and best serum creatinine concentration in dogs without resolution of azotemia.

Dogs	Initial creatinine (mg/dL)	Initial AKI grade	Best creatinine (mg/dL)^a^	Best CKD stage	Days to best creatinine
1	3.9	III	1.5	II	60
2	4.6	III	1.8	II	142
3	5.9	IV	2.1	II	86
4	3.9	III	1.5	II	104
5	4.4	III	1.8	II	53
6	4.0	III	2.3	II	207

Abbreviation: CKD, chronic kidney disease.

^a^ Not all dogs had best creatinine reassessed at the same time points throughout study.

### 3.6 Statistical outcomes

**FIGURE 1:**
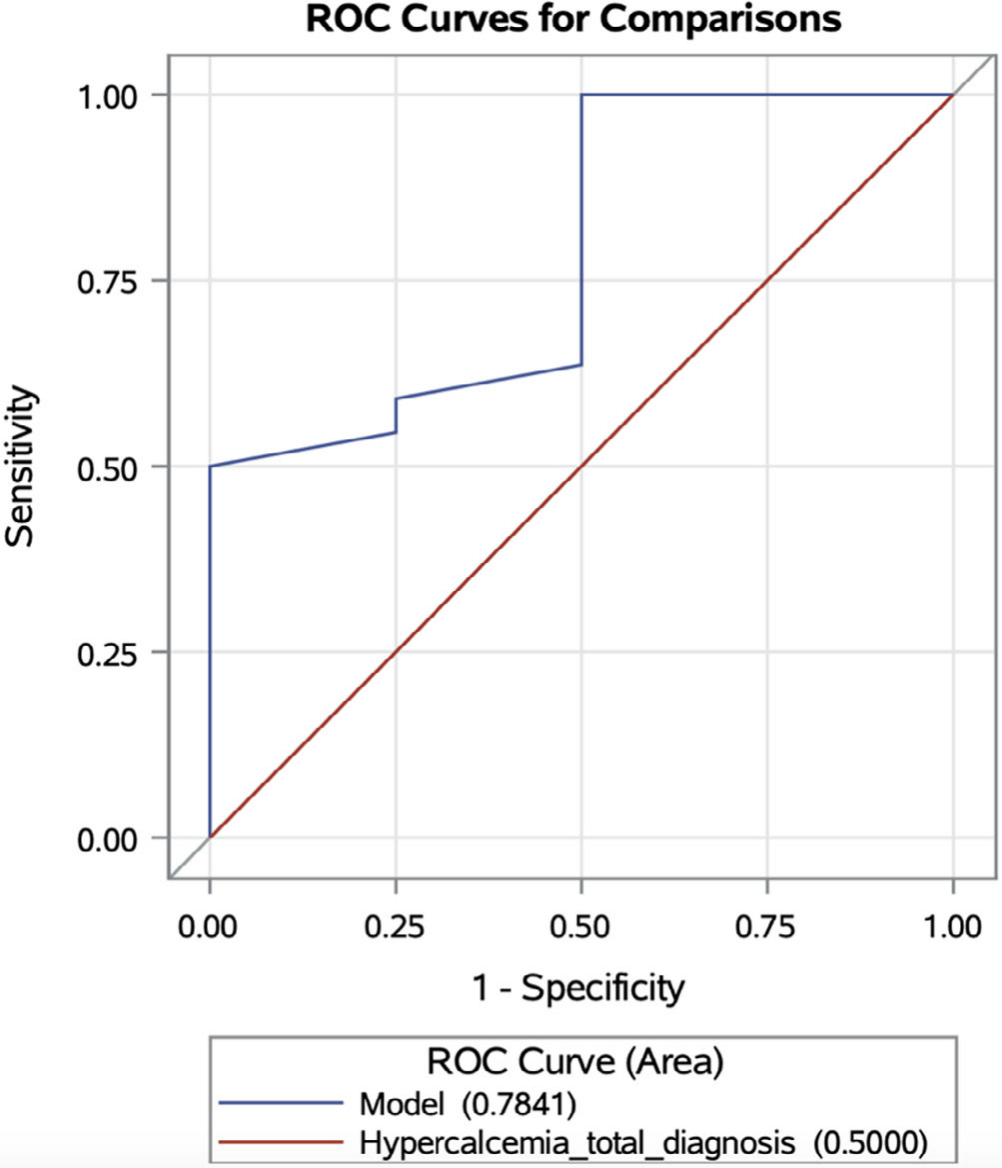
ROC curve for hypercalcemia. A cutoff of 17.5 mg/dL yielded a sensitivity of 100% and specificity of 50% for predicting resolution of azotemia.

While undergoing chemotherapy, 23/29 (79%) dogs achieved complete response (clinical remission of lymphoma), whereas 4/29 (14%) experienced a partial response and 2/29 (7%) had stable disease. On univariate analysis, IRIS grade III vs II, IRIS grade IV vs II, BUN concentration at diagnosis, serum creatinine concentration at diagnosis, hospitalization at diagnosis, sex, and serum total calcium concentration were assessed against resolution of azotemia. Baseline serum creatinine concentration (OR, 0.148; CI, 0.03‐0.734; *P* = .02) and total serum calcium (OR, 0.36; CI, 0.14‐0.93; *P* = .04) were significantly associated with resolution of azotemia whereas the remaining variables were not significant. Resolution of azotemia was 85% less likely for each 1‐unit increment in serum creatinine concentration (OR, 0.148; CI, 0.03‐0.734; *P* = .02). Resolution of azotemia was 64% less likely for each 1‐unit increase in serum total calcium concentration (OR, 0.36; CI, 0.14‐0.93; *P* = .04). On ROC analysis of serum total hypercalcemia, a cut‐off of 17.5 mg/dL showed 100% sensitivity and 50% specificity for predicting resolution of azotemia (Figure 1). On ROC analysis of serum creatinine concentration, a cut‐off of 3.0 mg/dL showed 91.3% sensitivity and 100% specificity for predicting resolution of azotemia.

### 3.7 Survival analysis

At time of data analysis, 1 dog remained alive, with last follow‐up confirming complete remission of lymphoma but development of renal disease at 995 days. This dog had resolution of azotemia after treatment at the time of lymphoma diagnosis. The remaining dogs were dead, with 20/29 (69%) having progressive lymphoma as the cause of death and 6/29 (20.7%) in remission from lymphoma at the time of death. One dog was euthanized during chemotherapy for an unknown reason, suspected to be non‐lymphoma‐related given the remission status at last follow‐up; the dog last received chemotherapy 4 days before euthanasia and was in complete remission at that visit. The remaining 2 dogs were lost to follow‐up, with times to follow‐up of 125 and 948 days, respectively. Both dogs lost to follow‐up did not have resolution of azotemia at their last assessment; 1 dog was in complete remission with hypercalcemia resolution whereas the other dog had lymphoma progression but was normocalcemic.

Death because of renal disease occurred in 4/26 (15%) dogs, with 2 dogs having concurrent lymphoma progression and worsening azotemia, 1 dying of a thrombus secondary to protein‐losing nephropathy (PLN) but in lymphoma remission based on necropsy results, and 1 dying of renal disease at an unknown date but in lymphoma remission at last follow‐up (1633 days). One dog had recurrent hypercalcemia documented at the time of progressive lymphoma and represented 1 of 2 dogs that died from concurrent lymphoma progression with worsening AKI. Of the 4 dogs dead because of renal disease, 3 did not have resolution of azotemia detected at diagnosis.

Three of the 7 dogs with renal changes detected on ultrasound examination died of renal‐associated causes, 2 of which had concurrent lymphoma progression and 1 that developed a thrombus secondary to PLN. There was no influence of the presence of renal ultrasound changes on disease‐related death (OR, 7.73; *P* = .46).

Two dogs died secondary to progressive lymphoma before 3 months; 1 at 30 days and 1 at 82 days. Both were included in statistical analysis because they underwent chemotherapy with an intended follow‐up of at least 3 months. The median survival time for 25/29 dogs was 247 days (95% CI, 157 to 314), censoring 2 dogs that were lost to follow‐up, 1 alive at time of data analysis (995 days), and 1 dead but with an unknown date of death (last follow‐up, 1633 days).

Survival time was not significantly impacted by severity of hypercalcemia or serum creatinine concentration at diagnosis or by resolution of azotemia.

### 3.8 Necropsy

Necropsies were performed on 3 dogs, with 2 dogs in lymphoma remission at death. Necropsy information for 1 dog confirmed lymphoma as the cause of death (follow‐up time of 30 days). Another was in remission at the time of euthanasia as a result of glossitis, suspected to be secondary to chemotherapy (follow‐up time, 248 days). This dog had received a lomustine‐based multi‐agent chemotherapy protocol as initial treatment for lymphoma followed by mechlorethamine, vincristine, procarbazine, and prednisone (MOPP) and dexamethasone, melphalan, actinomycin D, and cytosine arabinoside (DMAC) after subsequent relapses. Each of these dogs had no renal abnormalities detected at necropsy. The third dog was in lymphoma remission at death but succumbed to thrombi secondary to PLN (survival of 234 days). Although glomerular changes were detected on necropsy, the predominant feature detected in the kidneys was diffuse, marked, chronic interstitial fibrosis with tubular atrophy and degeneration. Of these 3 dogs, 2 initially had resolution of azotemia and the remaining dog did not (the dog that died of a thrombus secondary to PLN).

## 4 DISCUSSION

To our knowledge, ours is the first report evaluating long term outcomes in a group of dogs with intermediate‐ to high‐grade lymphoma with HM and concurrent azotemia that underwent treatment. Previous studies have found HM to be associated with other negative prognostic indicators (eg, T‐cell phenotype) in dogs with lymphoma rather than an independent negative prognostic indicator.^8^ Interestingly, 1 dog in our study was found to have B‐cell lymphoma based on IHC and PARR. Although finding a B‐cell immunophenotype is uncommon with paraneoplastic hypercalcemia, it has been reported previously by others but without clinical relevance.^19^, ^20^, ^21^ Furthermore, many dogs with HM are clinically ill (substage b) at presentation, which is considered a negative prognostic factor for outcome with the treatment of lymphoma.^8^ It is unclear if more intensive treatment would be beneficial for azotemic dogs with lymphoma as compared to those without azotemia. Furthermore, in our population of dogs, hospitalization did not significantly affect resolution of azotemia. Hypercalcemic nephropathy has been reported in dogs and can result from a variety of underlying disease processes.^14^ The effects of renal impairment associated with HM are multifactorial, specifically related to the effect of PTHrP on the response of collecting tubules to ADH and subsequent volume depletion, the direct toxic effect of calcium on the renal tubules, pre‐renal factors affecting GFR, and further decrease in GFR and vasoconstriction secondary to low parathyroid hormone concentration.^6^, ^22^ In humans, sustained hypercalcemia causing renal impairment can be mild and reversible with appropriate treatment and resolution of hypercalcemia or more severe and permanent, leading to marked renal damage.^22^

Despite literature to support the development of hypercalcemic nephropathy in dogs with underlying neoplasia, the prevalence and outcomes, especially in dogs with lymphoma have not been well described. One report described azotemia in hypercalcemic dogs, of which 71% had concurrent neoplasia, but the number of dogs included was small (n = 14).^23^ In another study evaluating 27 dogs with high‐grade mediastinal lymphoma, 48.1% of the hypercalcemic dogs were found to be azotemic, which represented 32.5% of all dogs in the study.^8^ Interestingly, hypercalcemia and azotemia did not affect median progression free survival or overall survival.^8^ Despite the prevalence of azotemia in this subset of dogs, the available literature has not documented azotemia as a negative prognostic indicator.^3^, ^8^

In our population, all dogs were classified as having substage b lymphoma, representing a clinical presentation indicating nonspecific illness. In humans with lymphoma, B‐symptoms are used to refer to lymphoma‐specific clinical signs whereas substage b, as it is referred to in dogs, is related to any clinical sign (with or without an association to lymphoma).^24^ Furthermore, historically, no established guidelines consistently determine clinical substage in dogs. Various factors have been used, with some studies relying on physical examination findings alone whereas others include changes such as hypercalcemia.^24^ A more standardized approach to classifying substage status in dogs exists, although limitations remain in the subjective nature of identifying both the clinical signs that contribute to substage and the severity that would classify a dog as substage b rather than a.^24^ Interestingly, in a recent study, treatment response was negatively impacted by both substage b and B‐symptoms, but more specifically B‐symptoms significantly decreased progression‐free survival time.^25^ That study indicated that generalized signs of illness may not have an impact on outcome of dogs with large cell B‐cell nodal lymphoma.^25^ Thus, it is difficult to elucidate the outcome in dogs ill from both nonspecific findings that may represent these B‐symptoms but also secondary illnesses, such as signs relating to hypercalcemia or azotemia. Likewise, in our population of dogs, 66% were hospitalized at the time of diagnosis, but the medical records did not clearly state if hospitalization was based on clinician recommendation after finding hypercalcemia and azotemia or a consequence of the dogs' substage b status. Although these dogs may be perceived as more ill than the general lymphoma population without HM and secondary azotemia, their overall outcome may not be impacted. However, data to support the clinical relevance of substage on outcome in this subset of dogs is still lacking.

Both the degree of increase in serum creatinine concentration and the severity of the serum total hypercalcemia at diagnosis were significantly associated with failure of resolution of azotemia. Moreover, the higher the serum total calcium concentration, the more likely it was that resolution of azotemia would not occur, although a serum total calcium concentration cut‐off of 17.5 mg/dL represented only 50% specificity. There may be merit suggesting that the higher the serum creatinine concentration, the more likely true renal impairment (ie, renal azotemia) is present rather than a substantial concurrent pre‐renal azotemia component, but there are no current reports in the literature to validate this hypothesis. Likewise, regardless of the underlying factor related to higher serum calcium concentration and less likelihood for resolution of azotemia, this result presents a clinically important factor to help guide clinicians in assessing the outcomes of this subset of dogs.

Two of the dogs in our study had received short‐term fluid therapy before referral. Neither dog had a USG documented until being evaluated at the referral hospital, at which time both were found to be isosthenuric. Although fluid therapy before assessment could have confounded our results, we included these 2 dogs because they were azotemic on repeat laboratory testing at the referral hospital despite having received fluid therapy, thus indicating the possibility of a renal component even if concurrent pre‐renal azotemia was present.

Abdominal ultrasound examination was performed in 17/29 (59%) dogs at baseline. Renal changes were detected in 7/17 (41%). Nonspecific changes that could be related to underlying renal impairment were present in 6/7 dogs, whereas 1/7 had bilateral pyelectasia, a finding that could be secondary to diuresis in the presence of normal renal function.^26^ Interestingly, only 3 dogs with renal changes ultimately died of disease related to renal injury, although 1 dog had a thrombus secondary to PLN and the other 2 also had concurrent progressive lymphoma. Renal ultrasound changes had no effect on disease‐related death. Renal medullary rim sign, which was present in 5/7 of our dogs, historically has been associated with pathologic renal lesions, although other possibilities exist, including idiopathic or toxin‐mediated acute tubular necrosis, pyogranulomatous vasculitis, chronic interstitial nephritis, or renal calcification secondary to hypercalcemia.^27^ More recent reports have described medullary rim sign in dogs without apparent renal pathology.^28^ Few reports have attempted to characterize canine hypercalcemic nephropathy in dogs with ultrasound changes. In humans, microscopic deposits of calcium in the renal parenchyma lead to a diffuse increase in echogenicity without acoustic shadowing whereas macroscopic calcification causes acoustic shadowing.^29^ These findings support the difficulty in ascribing discrete ultrasound abnormalities of the kidneys to hypercalcemia and support our findings, although in a small subset of dogs, death was not associated with renal changes detected on ultrasound examination.

Dogs in our study underwent chemotherapy treatment using different protocols, including lomustine‐based (single‐ or multi‐agent), COP, or CHOP‐based protocols. Because treatments were not standardized, statistical evaluation of type of chemotherapy protocol influencing outcome was not performed.^8^, ^30^

Interestingly, regardless of remission status of the lymphoma, all dogs had resolution of their hypercalcemia after starting treatment and the majority had resolution of azotemia. Normalization of serum calcium concentration is expected, because treatment should result in resolution of paraneoplastic hypercalcemia. An explanation for why some dogs had resolution of azotemia and others did not is unknown, including 2 dogs that remained at the same IRIS AKI grade and 4 dogs that had improved grades. Permanent renal impairment may occur in a subset of dogs regardless of overall outcome, and potential improvement in IRIS AKI grade may be related to a pre‐renal component (ie, dehydration). This could coincide with the significance of baseline serum creatinine concentration and serum total hypercalcemia and resolution of azotemia in our cohort of dogs. One dog in the cohort died of renal failure, specifically PLN. It is possible that this dog had pre‐existing PLN and that the azotemia that developed at the time of lymphoma diagnosis was pre‐renal. Additionally, PLN is a consequence of glomerular damage whereas HM causes tubular damage.^31^

Paraneoplastic syndromes can recur simultaneously at the time of cancer relapse without clinical recognition of the primary neoplasm.^1^ Although described in the literature, the likelihood of reoccurrence is not well described in dogs with lymphoma. In our population of dogs with progression of lymphoma, only 41% of those reassessed had recurrent hypercalcemia noted at the time of progression. Most dogs did not have recurrent azotemia at the time of lymphoma progression even with recurrent hypercalcemia. It remains unknown why only a subset of dogs develop recurrent hypercalcemia with lymphoma relapse with even fewer developing recurrent azotemia. The variability may be multifactorial in nature and include the frequency of re‐examination and calcium assessment as well as early detection of lymphoma relapse or mild recurrent hypercalcemia in dogs without clinical signs, and unknown factors.

Most dogs died secondary to progressive lymphoma. Only 4/26 (15.4%) of dogs died from renal disease, 2 of which also had concurrent lymphoma progression, thus confounding the likelihood of renal impairment as the predominant feature. Our intent was to include dogs treated for a minimum of 3 months based on likelihood for recovery from an AKI to occur. Based on our findings, the likelihood for renal failure causing death is low in this subset of dogs.

Our study had some limitations, including its design and small sample size, which decreased the overall power of the study.

When collecting data from the medical records, laboratory test results before development of lymphoma with hypercalcemia and azotemia were unavailable. This factor prohibited us from determining whether outcomes were influenced by pre‐existing renal disease. Based on the data collected, none of the dogs were known to have pre‐existing renal disease. Similarly, information available retrospectively in medical records for assessment of hydration status concurrent with renal function test results made it challenging to distinguish the potential impact of pre‐renal azotemia from inability to maximally concentrate urine secondary to the influence of calcium on renal concentrating ability.

Furthermore, few dogs were necropsied and antemortem renal biopsies were not performed in any of the dogs. Future studies including evaluation of renal histology should be considered to further assess dogs that fail to have resolution of azotemia after medical management and to evaluate permanent changes in renal architecture. Such results may provide more insight into the observation that, despite concurrent azotemia and renal impairment, death related to the azotemia is not likely.

## CONFLICT OF INTEREST DECLARATION

Authors declare no conflict of interest.

## OFF‐LABEL ANTIMICROBIAL DECLARATION

Authors declare no off‐label use of antimicrobials.

## INSTITUTIONAL ANIMAL CARE AND USE COMMITTEE (IACUC) OR OTHER APPROVAL DECLARATION

Authors declare no IACUC or other approval was needed.

## HUMAN ETHICS APPROVAL DECLARATION

Authors declare human ethics approval was not needed for this study.
